# Synergistic Activity of Second Mitochondrial-Derived Activator of Caspases Mimetic with Toll-like Receptor 8 Agonist Reverses HIV-1-Latency and Enhances Antiviral Immunity

**DOI:** 10.3390/ijms26062575

**Published:** 2025-03-13

**Authors:** Killian E. Vlaming, Jade Jansen, Godelieve J. de Bree, Neeltje A. Kootstra, Teunis B. H. Geijtenbeek

**Affiliations:** 1 Department of Experimental Immunology, Amsterdam UMC, University of Amsterdam, Meibergdreef 9, 1105 AZ Amsterdam, The Netherlands; 2 Amsterdam Institute for Immunology and Infectious Diseases, Meibergdreef 9, 1105 AZ Amsterdam, The Netherlands; 3 Department of Internal Medicine, Amsterdam UMC, University of Amsterdam, Meibergdreef 9, 1105 AZ Amsterdam, The Netherlands

**Keywords:** antiviral responses, SMAC mimetic, RIG-I like receptor (RLR), Toll-like receptor 8 (TLR8), HIV-1

## Abstract

HIV-1 infection is successfully treated by antiretroviral therapy; however, it is not curative as HIV-1 remains present in the viral reservoir. A strategy to eliminate the viral reservoir relies on the reactivation of the latent provirus to subsequently trigger immune-mediated clearance. Here, we investigated whether the activation of Toll-like receptor 8 (TLR8) or RIG-I-like receptor (RLR) together with the latency reversal agent (LRA) second mitochondrial-derived activator of caspases mimetics (SMACm) leads to HIV-1 reservoir reduction and antiviral immune activation. The TLR8 and RLR agonist elicited a robust pro-inflammatory cytokine response in PBMCs from both PWH and uninfected people. Notably, co-stimulation with SMACm specifically enhanced TLR8 induced pro-inflammatory cytokine as well as CD8 T cell responses. Ex vivo treatment of PBMCs from PWH with SMACm significantly decreased the size of the inducible HIV-1 reservoir, whereas targeting TLR8 or RLR reduced the HIV-1 reservoir in 50% of PWH ex vivo. Although co-stimulation with TLR8/RLR agonists further reduced the HIV-1 reservoir in 25% of PWH ex vivo, effectively inducing antiviral immunity may help eliminate reactivated HIV-1 cells in vivo. Our findings strongly suggest that LRAs can be used in combination with agonists for pattern recognition receptors to reactivate HIV-1 and induce antiviral immunity.

## 1. Introduction

While antiretroviral therapy (ART) has significantly improved the quality of life and life expectancy of people living with HIV-1 (PWH), it is not curative. The virus establishes a reservoir of cells harboring integrated HIV-1 DNA without active replication and viral protein production, thus evading immune detection [1]. The persistence of this viral reservoir is a major challenge for achieving a cure for HIV-1 [2].

Several approaches to developing a cure for HIV-1 are currently being investigated. Among these is the “shock and kill” method, which is designed to reactivate latent HIV-1, making cells sensitive to anti-HIV-1/apoptosis drugs, or antiviral immune responses [3,4]. Multiple latency-reversing agents (LRAs) have been identified for their ability to reactivate latent HIV-1 in vitro but achieving effective reservoir reduction in vivo remains challenging [5,6]. This is largely attributed to the lack of immune-mediated clearance [4,6]. Therefore, in addition to the combination of potent LRAs with apoptosis-inducing drugs, compounds that enhance antiviral immune responses might be required to fully eliminate HIV-1 reactivated cells over time.

Second, mitochondrial-derived activators of caspases mimetics (SMACm) are small molecules designed to inhibit inhibitors of apoptosis proteins (IAPs), which are often upregulated in HIV-1-infected cells to prevent apoptosis. By antagonizing IAPs, SMACm promotes apoptosis of infected cells [7]. In addition, SMACm activate the non-canonical NF-κB pathway (ncNF-κB), and to a lesser extent, the canonical pathway, both can activate HIV-1 transcription. This dual function of SMACm to reactivate and eliminate HIV-1 infected cells makes it a promising component of “shock and kill” strategies [8,9,10] and it has proven to be an effective LRA in preclinical animal models [11,12]. While apoptosis induction and reservoir reduction are observed, these effects remain incomplete and do not fully eliminate the viral reservoir.

Pattern recognition receptors (PRRs) such as Toll-like receptors (TLR) and RIG-I-like receptors (RLRs) recognize pathogen-associated molecular patterns (PAMPs) and are crucial for inducing immunity. TLR8 and RLRs RIG-I and MDA-5 are important in antiviral immunity. TLR8 is an endosomal receptor that recognizes single-stranded RNA and activates the canonical NF-κB pathway [13,14]. TLR8 activation in antigen-presenting cells leads to the induction of pro-inflammatory cytokines and type I interferons [15,16,17]. RIG-I and MDA5 activate Interferon Regulatory Factor (IRF) 3 and 7 as well as the canonical NF-κB pathway via mitochondrial antiviral signaling protein (MAVS) [18]. RLR therefore induces strong antiviral Interferon Stimulated Gene (ISG) responses as well as pro-inflammatory cytokines [19,20].

Here, we investigated whether the potent immune activator of TLR8 or RLR agonists, combined with SMACm, offer an effective two-pronged strategy to eliminate the HIV-1 reservoir by directly reactivating latent HIV-1 and boosting antiviral immunity to eliminate cells containing a reactivated HIV-1 provirus. Our data strongly suggest that the combination of a TLR8 agonist with SMACm, in particular, leads to a strong induction of a pro-inflammatory cytokine and CD8 T-cell response whilst also reducing the HIV-1 viral reservoir in peripheral blood mononuclear cells (PBMCs) from PWH.

## 2. Results

### 2.1. SMAC Mimetic Enhances TLR8-Induced Immune Responses

**Figure 1:**
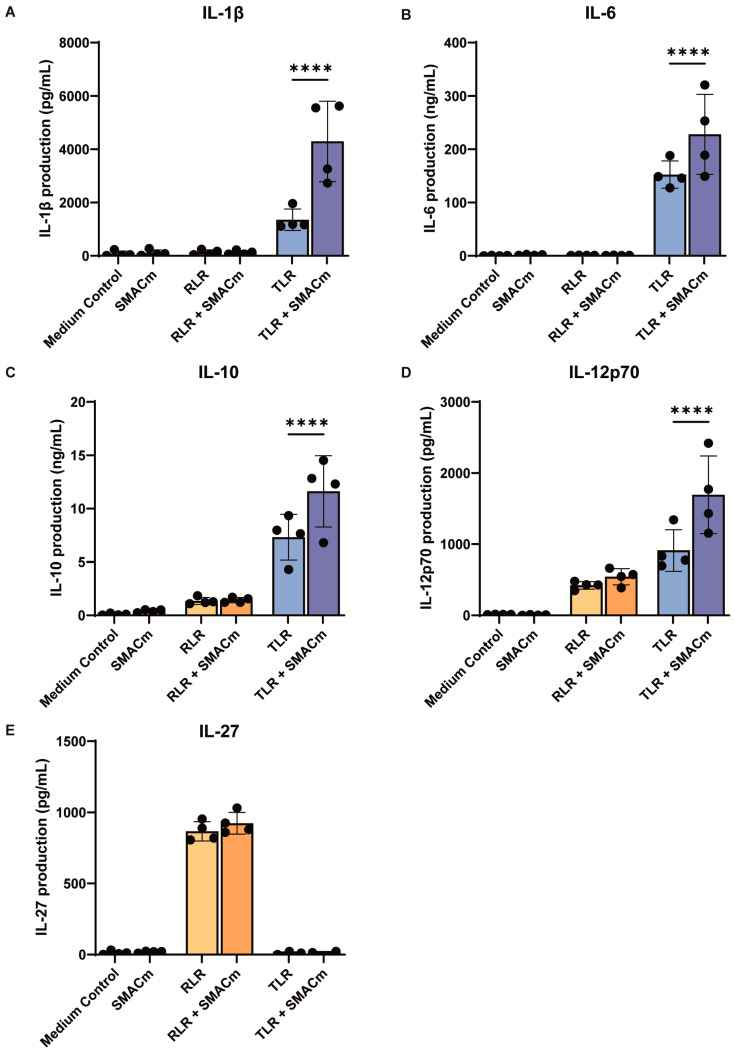
TLR8 and RLR, but not SMACm, induce cytokines in PBMCs. (**A**–**E**) PBMCs were stimulated for 24 h in the presence of RIG-I-like receptors (RLR) agonist, TLR8 agonist, or SMACm alone or in combination, and cytokines in the supernatant were measured by ELISA. Two-way ANOVA tests were performed to compare unpaired grouped data between donors. Statistical significance was set at, **** *p* < 0.0001.

We investigated the potency of the SMACm, TLR8, and RLR agonists AZD5582, GS9688, and Poly(I:C)-lyovec, respectively, to induce cytokine responses in PBMCs from uninfected individuals. PBMCs were stimulated with the compounds alone in combination and cytokine production was measured after 24 h. Treatment with SMACm did not induce cytokines, whereas TLR8 agonist-induced IL-1β, IL-6, IL-10, and IL-12p70, and RLR agonist-induced IL-10, IL-12p70 and IL-27 production (Figure 1A–E). Notably, SMACm co-stimulation with the TLR8 agonist enhanced the production of IL-1β, IL-6, IL-10, and IL-12p70 compared to that by TLR8 alone (Figure 1A–D). SMACm did not affect RLR-induced cytokine production (Figure 1E). These data suggest that SMACm enhances TLR8- but not RLR-induced cytokine responses.

### 2.2. SMACm Enhances TLR8-Induced Cytotoxic T Cell Activity

**Figure 2:**
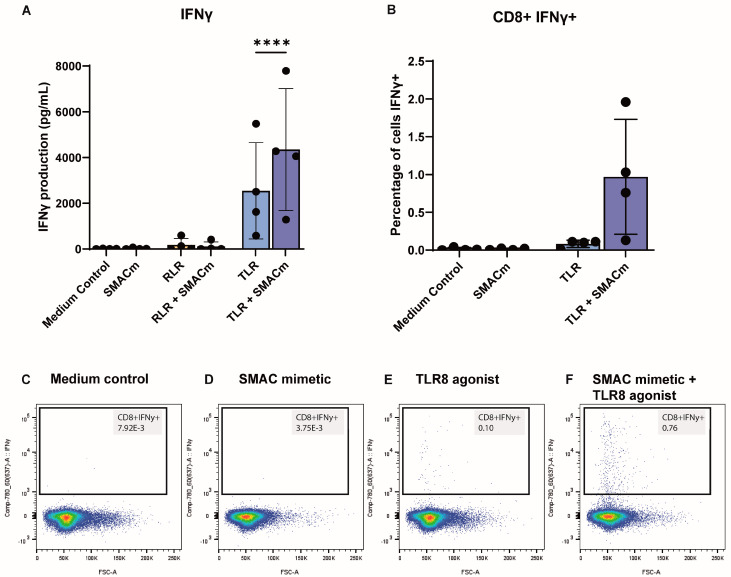
SMACm enhanced TLR8−induced IFNγ secretion by uninfected PBMCs. (**A**) PBMCs were stimulated for 24 h with TLR8, RLR agonist, and SMACm alone or in combination and IFNγ levels were measured by ELISA; (**B**) intracellular IFNγ analysis was performed following stimulation with the compounds for 18 h; (**C**–**F**) individual flowcytometry plots showing intracellular IFNγ staining in CD3+CD8+ T cells unstimulated or treated with different compounds. Colors in flowcytometry dotplot are related to cell density with a color scale from blue to red corresponding to low to high cell density. Statistical significance was set at **** *p* < 0.0001.

The induction of IFNγ-producing cytotoxic T cells is paramount to an effective antiviral immune response. We therefore analyzed whether SMACm, TLR8, or RLR agonists alone or in combination enhanced cytotoxic T-cell responses. TLR8 agonist alone induced potent expression of IFNγ in contrast to SMACm and RLR agonist alone (Figure 2A). Notably, co-stimulation of TLR8 agonist with SMACm induced a significant, almost 2-fold increase, in IFNγ production compared to TLR8 agonist alone (Figure 2A). Intracellular staining for IFNγ showed that TLR8 agonist, but not SMACm alone, induced IFNγ induction in CD8+ T cells compared to the medium control (Figure 2B,C and Figure S1). Notably, co-stimulation of TLR8 agonist with SMACm increased the number of IFNγ producing CD3+CD8+ T cells (Figure 2B,C). Thus, the combination of SMACm with TLR8 agonists induced more effective cytotoxic T-cell responses compared to SMACm and TLR8 agonists alone.

### 2.3. SMAC Mimetic Modulates TLR8 Mediated Immune Activation in PWH

**Figure 3:**
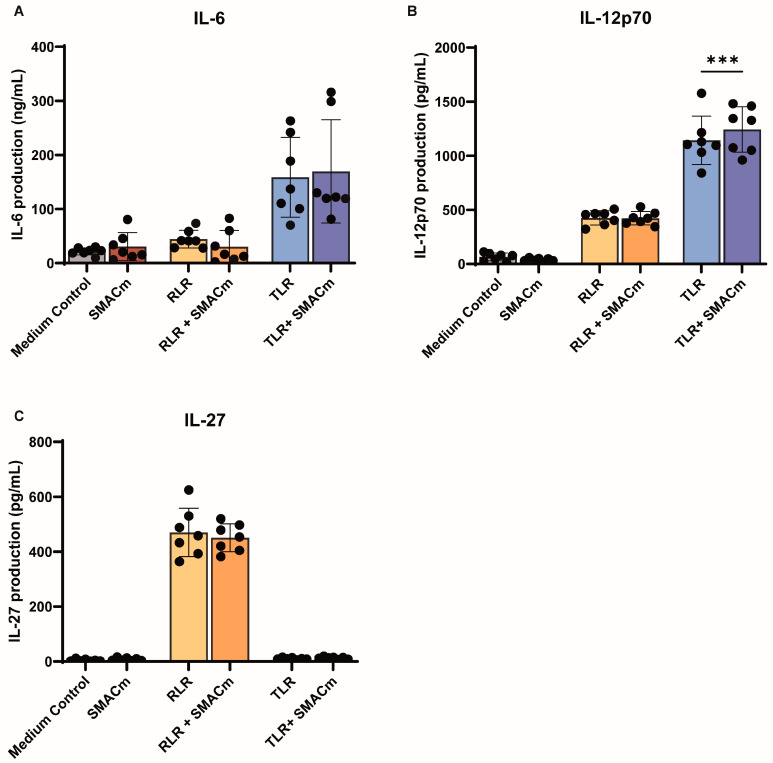
SMAC mimetic influences TLR8−driven immune activation in PBMCs of PWH. (**A**–**C**) PBMCs were stimulated for 24 h in the presence of RLR-, TLR8 agonist, or SMACm alone or in combination, and cytokines were measured by ELISA. Two-way ANOVA tests were performed to compare unpaired grouped data between donors. Statistical significance was set at *** *p* < 0.001.

Next, we investigated the effect of the compounds on PBMCs from seven participants from the Amsterdam Cohort Studies for HIV/AIDS (ACS) who had an untreated course of HIV-1 infection (Table 1). PBMCs were treated with the compounds for 24 h. Unstimulated PBMCs from PWH had higher levels of IL-6 and IL-12p70 compared to those from uninfected individuals (Figure 3A,B). SMACm treatment alone did not induce cytokine production, whereas treatment with the TLR8 induced IL-6 and IL-12p70 and RLR induced IL-12p70 and IL-27 (Figure 3A–C). Co-stimulation with SMACm and TLR8 significantly increased IL-12p70 compared to TLR8 alone, albeit lower as observed for uninfected individuals (Figure 1D and Figure 3B). SMACm did not affect RLR-induced cytokine response. These data suggest that SMACm enhances TLR8-induced IL-12p70 production in PBMCs from PWH but less efficiently than observed in uninfected individuals.

### 2.4. TLR8 and RLR Agonists Do Not Alter SMACm-Induced Reservoir Reduction

As previously demonstrated, SMACm effectively reduces the HIV-1 reservoir ex vivo [8]. We, therefore, evaluated whether TLR8 and RLR agonists alone or in combination with SMACm could modulate reservoir reduction in PBMC from PWH. We determined the inducible HIV-1 reservoir ex vivo in four of the ACS participants (Table 1) using the inducible HIV-1 reservoir reduction assay (HIVRRA) [8], which measures the reactivation of the replication-competent viral reservoir.

**Figure 4:**
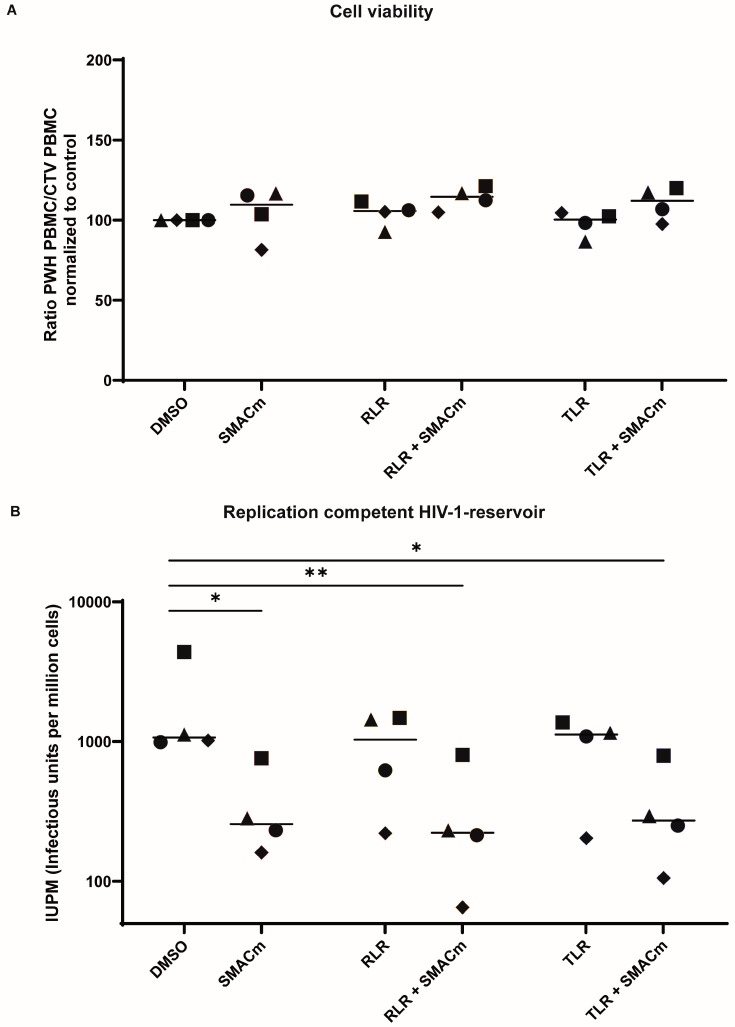
The inducible HIV-1 reservoir in PBMCs from PWH is reduced upon treatment with TLR8 or RLR agonist and SMACm. (**A**) Ratio PWH PBMCs/uninfected cell trace violet (CTV) stained PBMCs normalized to DMSO are shown to analyze cell survival after compound treatment; the median is displayed. (**B**) Median infectious units per million cells (IUPM) after treatment is displayed; uncorrected Dunn’s test was performed to compare paired data between treatment groups and DMSO control. Symbols indicate results obtained using PBMC from the same PWH. Statistical significance was set at * *p* < 0.05, ** *p* < 0.01.

The inducible HIV-1 reservoir is expressed as infectious units per million cells (IUPM). Using a logistic regression analysis (Figure S2), the IUPM was calculated for each condition for every participant. To determine the specificity of the compounds to eliminate HIV-1 infected cells, total PWH PBMC numbers were analyzed after treatment with the different compounds and combinations. In every condition, the proportion of PWH PBMCs did not decrease compared to CTV-stained PBMCs, indicating that SMACm, TLR8, and RLR agonists alone or in combination did not affect uninfected cells (Figure 4A).

The median inducible HIV-1 reservoir size measured in the unstimulated (medium) control was 1071 IUPM (range 989–4383) (Table 2 and Figure 4B). SMACm treatment of the PBMC from the PWH significantly reduced the inducible HIV-1 reservoir size (median: 257 IUPM, range: 161–761) compared to the untreated control (Figure 4B). Treatment with either RLR or TLR8 agonist alone did not have a significant impact on the size of the inducible HIV-1 reservoir control (TLR8 agonist median IUPM 1089, range: 204–1371) (RLR agonist median IUPM: 1033, range: 221–1480) (Figure 4B). Interestingly, a reduction in the HIV-1 reservoir was observed for two participants in the TLR8 agonist- and three participants in the RLR agonist-treated groups (Figure 4B), suggesting that their effectiveness is participant-specific and may depend on either the mechanisms of HIV-1 latency or anti-HIV-1 immune responses. Notably, combining SMACm with TLR8 agonist and/or RLR agonist did not interfere with the capacity of SMACm to reduce the size of the viral reservoir and even enhanced reservoir reduction in one donor (TLR8 agonist + SMACm median IUPM: 272, range 106–793) (RLR agonist + SMACm median IUPM: 223, range: 65–801) (Figure 4B). These data suggest that SMACm strongly reduces the HIV-1 reservoir and can be enhanced by RLR and TLR8 co-stimulation in some cases. Moreover, as these compounds did not induce cell death in total PBMCs, meaning no effect on uninfected cells, our findings suggest a disproportionate impact on cells harboring HIV-1 reservoirs.

## 3. Discussion

Although multiple LRAs can reactivate latent HIV-1 in vitro, effective reservoir reduction in vivo remains challenging due to insufficient clearance of infected cells [5]. This highlights the need for new therapeutic options. Strong immune-modulating agents that stimulate antiviral immune responses to eliminate reactivated cells could be effective when combined with LRAs. In this study, we examined the effects of a SMACm in combination with two different immunomodulatory compounds, TLR8 or RLR agonist, on cytokine induction, cytotoxic T-cell responses, and reduction in the HIV-1 reservoir. Our findings strongly suggest that the combination of a TLR8 agonist with an SMACm induces a robust pro-inflammatory cytokine profile as well as cytotoxic T-cell response in PBMC from uninfected individuals. Similarly, TLR8 agonists with SMACm also induced a pro-inflammatory cytokine response in PBMC from PWH, albeit to a lesser extent, likely due to immune dysfunction observed with chronic infection in untreated PWH. SMACm consistently reduced the replication-competent HIV-1 reservoir in all individuals. This reduction was not affected by the presence of TLR8 or RLR agonists. Moreover, depending on the individual, we observed reservoir reductions with TLR8 and RLR stimulation. This variable effect of TLR8 and RLR activation may be due to individual specific differences in the composition of the viral reservoir resulting in the reactivation of only part of the reservoir due to different mechanisms of viral latency. Moreover, the condition of the immune system may differ between individuals and affect cytokine responses as well as subsequent NK- and CD8+ T-cell activation. TLR8 and RLR activation does not interfere with reservoir reduction by SMACm, indicating the potential of combining SMACm and immune agonists to achieve immune control while depleting HIV reservoirs.

SMACm functions via the degradation of the inhibitor of apoptosis proteins (IAPs). These IAPs are also involved in pro-inflammatory pathways, such as the NF-κB pathway, utilized by both TLR8 and RLR [21,22]. Where both TLR8 and RLR predominantly utilize the canonical NF-κB pathway, characterized by RelA and p50 subunits, SMACm activates the non-canonical NF-κB, subunits RelB, and p52 [21,22]. SMACm induced no cytokine response in PBMCs, indicating that SMACm is not an effective activator of the innate immune system. Treatment of uninfected PBMCs with a TLR8 agonist resulted in a robust induction of pro-inflammatory cytokines. The RLR agonist induced a different cytokine profile, typified by the presence of the ISG IL-27 [19,23,24,25]. Collectively, the cytokines induced by the TLR8 and RLR agonist boost the ability of cytotoxic T lymphocytes (CTLs) to recognize and eliminate HIV-infected cells, enhancing overall antiviral responses [19,20]. Pro-inflammatory cytokine IL-12p70 plays a crucial role in initiating adaptive immune responses against viruses by promoting T helper type 1 differentiation and activation of NK and CTLs [26]. Additionally, the IFN-stimulated gene IL-27 has been identified as a key factor in enhancing CD8 T-cell responses and facilitating the development of follicular T helper cells [27,28]. We observed crosstalk between TLR8 agonists and SMACm leading to synergy and potent induction of cytokines. Combining TLR8 agonists with SMACm resulted in even higher levels of IL-1β, IL-6, IL-10, and IL-12p70 in PBMCs, suggesting interplay between the two NF-κB pathways. The additive effect of the SMACm points to the modulation of RelB on RelA activity as SMACm activates both RelB and RelA. Interestingly, RelB has been implicated in the suppression of immunity [29]; however, RelB has also been described to enhance the transcription of pro-inflammatory cytokines induced by RelA [30]. This suggests RelB has pleiotropic functions, allowing for specific tailoring of immune responses.

Cytotoxic T cells (CD8+ T cells) play a vital role in clearing viral reservoirs. In our study, we observed a robust induction of IFNγ upon co-stimulation with SMACm and TLR8 agonists. IFNγ is a crucial cytokine for antiviral immunity, enhancing the cytotoxic activity of CD8+ T cells and natural killer cells, and promoting antigen presentation [31]. The increased activation of cytotoxic T cells by a combination of TLR8 and SMACm could be due to enhanced expression of IL-12p70, which we also observed. IL-12p70 is known to enhance the production of IFNγ by T helper 1 cells as well as directly enhance the function of CTLs themselves [32,33]. Furthermore, IL-1β enhanced CD8+ T cell effector functionality [34]. TLR8 stimulation in monocytes and DCs leads to the production of pro-inflammatory cytokines and type I interferons, which can enhance the activation and proliferation of T cells [20,35]. The increased production of IFNγ suggests that combining SMACm with TLR8 agonist further enhances anti-HIV-1 T cell immunity.

Even though TLR8 and RLR induced cytokines in PBMCs from untreated PWH, compared to HIV-uninfected PBMCs this effect was dampened. The expression of IL-6, IL-12p70, and IL-27 was induced but at a lower level after TLR8 or RLR agonist treatment. Following the co-stimulation of the TLR8 agonist and SMACm, an enhanced IL-12p70 response was observed but less strongly than observed for uninfected PBMCs. Persistent immune activation and inflammation are hallmarks of immune dysfunction in chronic HIV-1 infection [36,37,38,39,40,41,42,43,44,45,46,47]. Consistent with this notion, we observed low levels of IL-6 and IL-12p70 secretion in untreated PBMCs from PWH, which was not observed in PBMCs from uninfected donors. Although HIV-1-associated immune dysfunction in PWH is associated with less effective induction of pro-inflammatory cytokines in our assays, we show that the combination of TLR8 and SMACm still has the potential to induce immune responses in PBMCs from PWH, albeit reduced as compared to uninfected donors. IL-12p70, IL-27, and IFNγ each enhance immunity through distinct pathways, activating cellular NK cell and CD8+ T cell-mediated responses to clear infected cells. Our findings demonstrate that the induction of these cytokines can be synergistically increased by combining SMACm with TLR8 agonists, even in PBMC from PWH albeit to a lesser extent due to HIV-associated immune dysfunction. Indeed, PWH selected for this study is untreated, showing that even in PWH with a more severe immune deficiency, pro-inflammatory cytokine production can be induced.

The HIV-1 reservoir is known to be multi-faceted, consisting of both replication competent and defective proviruses. The inducible replication-competent provirus is responsible for viral rebound upon ART interruption and HIV-1 persistence. With the HIVRRA, we measure this part of the reservoir through infection of reporter TZM-BL cells, thus confirming reactivation of infectious full-length virus. Stimulating PBMCs from PWH with SMACm, TLR8-, or RLR agonists revealed a participant-dependent reduction in the replication-competent reservoir across all compounds, with SMACm displaying the most consistent, and potent, reduction even when combined with the TLR8 or RLR agonists. Our results align with the existing literature, which supports the role of IAP inhibitors in enhancing cell death for eliminating HIV-1-infected cells [8,48,49]. In addition, we show that SMACm alone does not induce a broad cytokine response, yet potently enhanced TLR8 mediated immunity to improve control following reservoir depletion.

The TLR8 and RLR agonists alone also demonstrated reservoir reduction without bystander cytotoxicity, albeit variable between individuals. This suggests that these compounds may potentially reactivate part of the viral reservoir dependent on the mechanism of viral latency and co-stimulation with SMACm. The subsequent reduction in the viral reservoir can be a direct consequence of the viral reactivation or is immune-mediated as cytokine production as well as NK and CD8+ T-cell responses will be activated. As TLR8 and RLR agonists alone and in combination with SMACm activate different immune pathways, we propose that multiple mechanisms drive the observed reservoir reduction. Moreover, differences in PWH with regard to the viral reservoir, including diverse mechanisms underlying latency [50,51,52,53,54,55] and the level of HIV immune dysfunction may explain the variable effects of TLR8 and RLR agonists on the viral reservoir.

These findings highlight the potential of combining multiple compounds that target different pathways to eradicate the HIV-1 reservoir. Of particular note, here is the specific cytotoxicity to infected cells this combination exhibited, which emphasizes the feasibility of these strategies. While these data provide a proof of concept, a key limitation is the ex vivo nature of this research. PBMC cultures provide valuable insight into reservoir clearance and immune induction, but this does not reflect the reservoir in PWH residing in different tissues. Therefore, these results are to be considered preliminary and in need of further exploration in translational and clinical research. Moreover, reduction in the viral reservoir was only studied in PBMC from men diagnosed with HIV-1 who have sex with men. Further research should include participants across diverse populations of PWH to ensure the generalizability of the results.

TLR8 agonists and SMAC mimetics have been validated for their safety profile, being well tolerated in vivo [56,57]. Given the cytokine profile observed in our study, particular attention should be given to the risk of excessive immune activation in future trials, particularly as the strongest effects were observed when SMACm and TLR8 agonists were combined. In order to achieve an HIV-1 cure, the viral reservoir needs to be eliminated, which has proven to be difficult. Therefore, a strong reduction and subsequent effective control by antiviral immunity would provide the basis for a functional cure [58]. SMACm alone effectively reduced the HIV-1 reservoir in PBMCs from all four PWHs ex vivo. Although the combination of SMACm and the TLR8 agonist did not result in a greater reduction in the reservoir compared to SMACm alone, with further reduction observed in only one of four samples, the combination did induce a strong adaptive immune response. This suggests that combining SMACm with the TLR8 agonist may be effective for in vivo reservoir elimination by reducing the viral reservoir as well as inducing antiviral adaptive immunity to control HIV-1 infection over time, thereby further decreasing the HIV-1 reservoir.

## 4. Materials and Methods

### 4.1. Study Participants

The Amsterdam Cohort Studies (ACS) on HIV infection and AIDS is a prospective study among men who have sex with men and injecting drug users that started in 1984 [59]. Seven participants diagnosed with HIV-1 who had an untreated course of infection were selected for this study (Table 1).

Control samples were obtained through the Dutch National Blood Bank in Amsterdam, The Netherlands (Sanquin).

### 4.2. Compounds

SMACm AZD5582 (Tebu-Bio, Le Perray en Yvelines, France) was dissolved in DMSO at a concentration of 10 mM and stored at −80 °C. Prior to use, AZD5582 was diluted in culture medium to a final concentration of 0.02 μM.

TLR8 agonist GS9688 (Medchemexpress, Monmouth Junction, NJ, USA) was dissolved in DMSO at a concentration of 10 mM and stored at −20 °C. Prior to use, GS9688 was diluted down to a working stock of 1 mM in demi-water and subsequently further diluted in culture medium to a final concentration of 1 μM.

RLR agonist Poly(I:C)-lyovec (Invivogen, Toulouse, France) was reconstituted in demi-water to a concentration of 50 μg/mL as per the manufacturer’s instructions. It was subsequently further diluted in culture medium to a final concentration of 2 μg/mL.

### 4.3. Cytokine Secretion

Control PBMCs were cultured in Roswell Park Memorial Institute (RPMI) (ThermoFisher Scientific, Waltham, MA, USA) medium enriched with 10% Fetal calf serum (FCS) (HyClone, Cytiva, Marlborough, MA, USA), 100 U/mL penicillin (ThermoFisher Scientific), 100 µg/mL streptomycin (ThermoFisher Scientific), 2 mML-glutamine (Lonza, Basel, Switzerland), and 20 U/mL IL-2 (Miltenyi Biotec, Bergisch Gladbach, Germany).

PBMCs were stimulated for 24 h with the compounds and subsequently supernatant was harvested. Secretion of IL-6, IL-10, IL-12p70, IL-27 was measured by ELISA as described by the manufacturer (IL-1β, IL-6, IL-10, IL-12p70 (eBiosciences Waltham, MA, USA), IL-27 (uCy-tech, Utrecht, The Netherlands)). OD450 nm values were measured using BioTek Synergy HT.

For IFNγ determination, PBMCs were initially cultured for 24 h with the compounds, after which the supernatant was harvested and analyzed by ELISA (Human IFN-gamma DuoSet ELISA, R&D Systems, Minneapolis, MN, USA).

### 4.4. Inducible HIV-1 Reservoir Reduction Assay (HIVRRA)

PBMCs from PWH were cultured in Iscove’s Modified Dulbecco’s Medium (IMDM) (ThermoFisher Scientific) supplemented with 10% FCS, antibiotics (100 U/mL penicillin, 100 µg/mL streptomycin and ciproxine 5 µg/mL) and 20 U/mL IL-2. PBMCs were cultured at 37 °C in a humidified 5% CO_2_ incubator.

TZM-BL cells, which contain an HIV-1 long terminal repeat (LTR) driven luciferase gene, [60] were obtained through the NIH HIV Reagent Program, Division of AIDS, NIAID, NIH, and were cultured in IMDM supplemented with 10% FCS and antibiotics (100 U/mL penicillin and 100 μg/mL streptomycin) at 37 °C in a humidified 10% CO_2_ incubator.

The frequency of inducible HIV-1-infected CD4+ T cells from chronic PWH was determined by the HIVRRA [8]. In summary, PBMCs from PWH were exposed to 0.02 μM SMACm, 2 µg/mL RLR agonist, or 1 μM TLR8 agonist, alone or in combination with SMACm for two days in the presence of 10 μM Saquinavir. Following this, PBMCs were stimulated with 1 μg/mL PHA in the presence of Saquinavir. After two days, PBMCs were washed and seeded in a 4-fold titration containing 3 × 10^4^ PBMCs in the first row and serially diluted 1:2 across a 96-well plate onto 2 × 10^4^ TZM-BL cells. After four days, the luciferase activity, driven by the HIV-1 LTR, was quantified in TZM-BL cells co-cultured with PBMCs from PWH by the addition of 25 μL luciferase activity reagent (LAR) substrate (0.83 mM ATP, 0.83 mM of d-Luciferin (Duchefa Biochemie B.V., Haarlem, The Netherlands), 18.7 mM MgCl_2_, 0.78 μM Na_2_H_2_P_2_O_7_, 38.9 mM Tris (pH 7.8), 0.39% glycerol, 0.03% Triton X-100 and 2.6 μM dithiothreitol) and measuring luminescence in relative light units (RLU) using a luminometer (Berthold Technologies, Bad Wildbad, Germany). The calculation of relative IUPM was based on 30% of the maximum RLU using logistic regression and is a measure for the functional HIV-1 reservoir (Figure S2).

To evaluate the cytotoxicity of the compounds during the assay, a consistent amount of CTV-labeled PBMCs from blood donors were used as spike-in and the ratio between CTV-labeled PBMC and unlabeled PWH PBMC was determined by flowcytometry. This also allowed us to adjust for PWH PBMC input for subsequent reservoir calculations.

### 4.5. Flowcytometry

HIV-negative PBMCs utilized in the HIVRRA were cultured as described in 4.4, then stained with CellTrace™ Violet (Thermo Fisher Scientific) according to the manufacturer’s protocol and fixed using BD CellFIX™ (BD Biosciences, Franklin Lakes, NJ, USA) afterward. Fluorescence was measured with the BD FACSCanto II (BD Biosciences).

For intracellular IFNγ analysis, PBMCs were cultured in a medium as described in 4.3, cultured for 18 h with SMACm, TLR8 agonist, or RLR agonist, alone or in combination with SMACm, after which BD GolgiPlug (containing Breveldin A) or BD GolgiStop (containing monensin) (both BD Biosciences), was added for an additional 6 h. Subsequently, cells were fixed using BD Cytofix (BD Biosciences) as described by the manufacturer. Cells were permeabilized using BD Perm/Wash buffer (BD Biosciences) for 15 min prior to being stained using BV480 conjugated anti-CD3 (1:250, Biolegend, San Diego, CA, USA), BB515 conjugated anti-CD4 (1:250, BD Biosciences), BV785 conjugated anti-CD8 (1:500, Biolegend) BV711 conjugated anti-CD14 (1:250, Biolegend), BV421 conjugated anti-CD16 (1:250, Biolegend), PE-conjugated anti-CD56 (1:125, Biolegend) in the presence of BD Perm/Wash buffer. Fluorescence was measured on a BD Symphony A1 (BD Biosciences). Flowcytometry data were analyzed using FlowJo version 10 (Treestar, Ashland, OR, USA).

### 4.6. Statistical Analysis

Statistical analysis of obtained data was performed using Graphpad Prism 10 (Graphpad Prism, San Diego, USA). One-way and Two-way ANOVA tests were performed to compare unpaired grouped data between donors, or paired within donors (IL-1β, IL-6, IL-10, IL-12p70, IL-27 Elisa). An uncorrected Dunn’s test was performed to compare paired data of the HIVRRA between treated and control conditions.

## Figures and Tables

**Table 1:** Participant characteristics.

Characteristic	Value
Total participants	7
Gender: male	100%
Age at time of sampling (years, range)	38 (27–52)
CD4+T cell count (cells/mm^3^, range)	521 (180–800)
Plasma viral load (copies/mL, range)	21,012 (2650–34,000)

**Table 2:** Mean and median IUPM from HIVRRA after treatment with different conditions.

Condition	Median IUPM	Mean IUPM	Range
Medium control	1071	1879	989–4383
SMACm	257	359	161–761
TLR	1089	955	204–1371
TLR + SMACm	272	361	106–793
RLR	1033	941	221–1480
RLR + SMACm	223	328	65–801

## Data Availability

The original contributions presented in this study are included in the article/Supplementary Materials. The raw data supporting the conclusions of this article will be made available by the authors upon reasonable request.

## Supplementary Materials

The following supporting information can be downloaded at: https://www.mdpi.com/article/10.3390/ijms26062575/s1.

## Abbreviations

The following abbreviations are used in this manuscript:

HIV-1	Human Immunodeficiency Virus 1
SMACm	Second mitochondrial-derived activator of caspases mimetics
LRA	Latency reversal agent
TLR8	Toll-like receptor 8
RLR	Rig-I-like receptor
PBMC	Peripheral blood mononuclear cells
ART	Antiretroviral therapy
NF-κB	Nuclear factor kappa-light-chain-enhancer of activated B cells
PRR	Pattern recognition receptor
PAMP	Pathogen-associated molecular patterns
RIG-I	Retinoic acid-inducible gene I
MDA5	Melanoma differentiation-associated protein 5
CTL	Cytotoxic T lymphocytes
MAVS	Mitochondrial antiviral-signaling protein
IRF	Interferon regulatory factor
PWH	People living with HIV
ACS	The Amsterdam Cohort Studies
LTR	Long terminal repeat
FCS	Fetal calf serum
IFNγ	Interferon gamma
RLU	Relative light units
CTV	Cell trace violet
HIVRRA	Inducible HIV-1 reservoir reduction assay
DMSO	Dimethylsulfoxide
IUPM	Infectious units per million cells
NK	Natural killer cells
TNF-α	Tumor necrosis factor
LPS	Lipopolysaccharides
AIDS	Acquired immunodeficiency syndrome
IMDM	Iscove’s Modified Dulbecco’s Medium
RPMI	Roswell Park Memorial Institute
