# Supplementary material for: Synergistic Activity of Second Mitochondrial-Derived Activator of Caspases Mimetic with Toll-like Receptor 8 Agonist Reverses HIV-1-Latency and Enhances Antiviral Immunity
Source: Int J Mol Sci. 2025 Mar 13;26(6):2575. doi: 10.3390/ijms26062575 (PMC11941979; doi:10.3390/ijms26062575)
Supplement: Supplementary file 1 [file ijms-26-02575-s001.zip › ijms-3504526-supplementary.pdf]

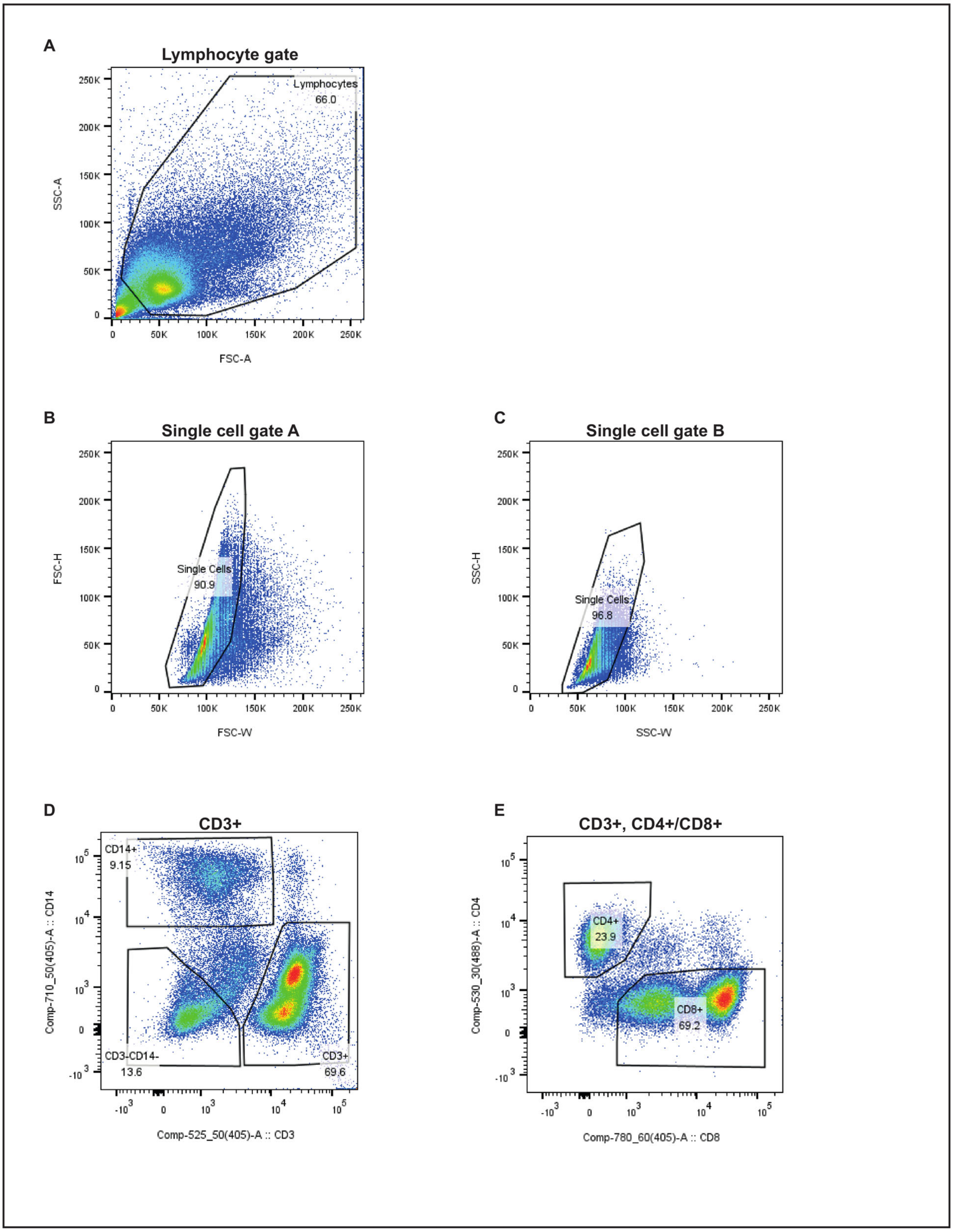

Figure S1. Gating strategy intracellular IFNy staining. Colors in flowcytometry dotplot are related to cell density with a color scale from blue to red cor-responding to low to high cell density.

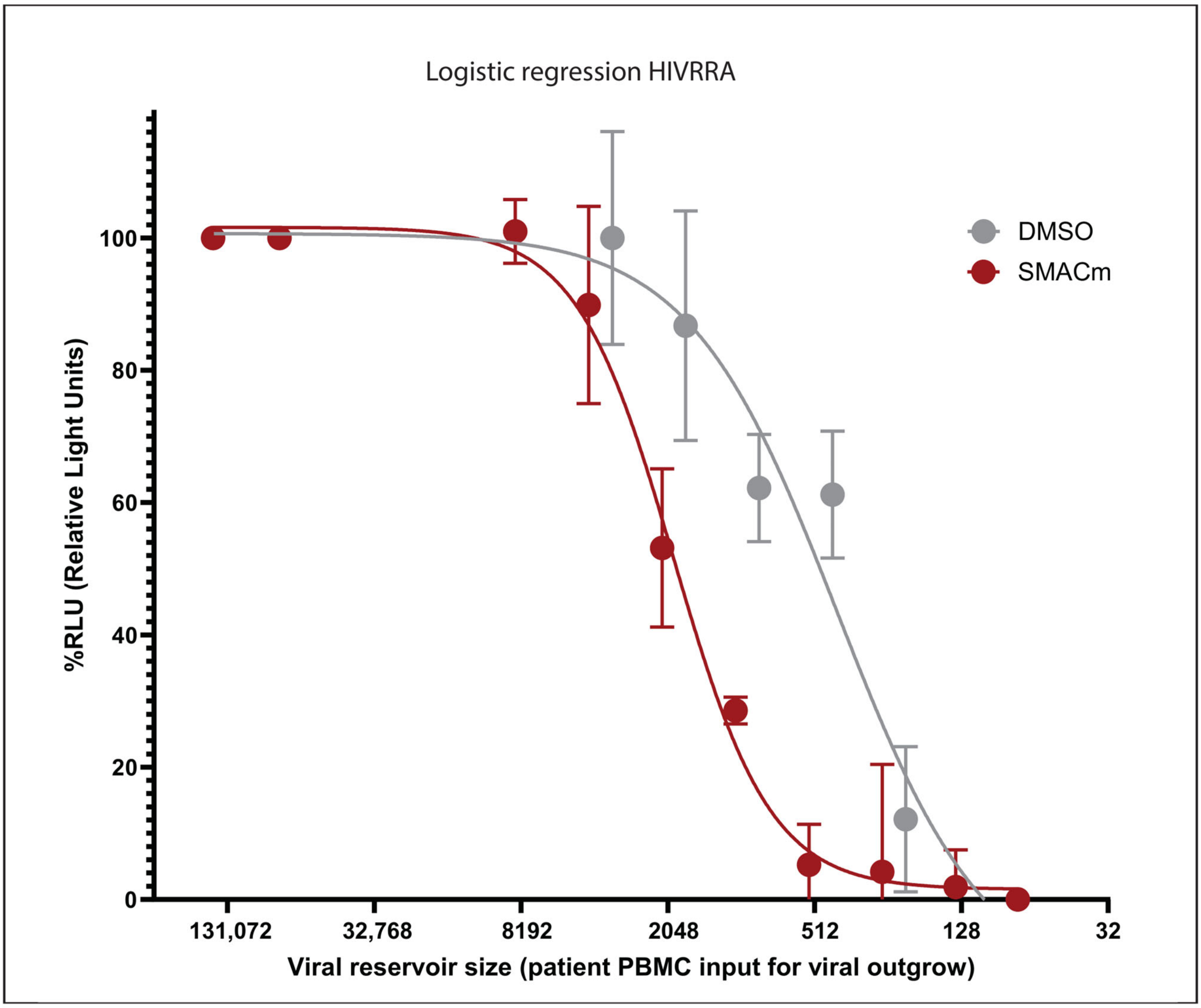

Figure S2. Patient 5, example log regression HIVRRA.
